# The Treatment of Functional Contracture by Fatigue

**Published:** 1918

**Authors:** 


***The Treatment of Functional Contracture by Fatigue.*** By E. F. Reeve, M. B., B. S. Lond., M. R. C. S., L. R. C. P. Lond., Captain (Temporary) R. A. M. C.

The principle of the treatment consists in producing a condition of fatigue in the muscles responsible for the contracture. This is obtained by continuous passive movements in a direction opposed to the normal action of the contracted muscles.

Description in detail of eight cases shows that most of the patients had been suffering from contractures for a great length of time. The following is an example : —

W. H., aged 37, was knocked over by a bursting shell in September, 1916. For some months he was unable to walk or even to stand; there were general tremors, and his left foot was fixed in a position of marked flexion. On admission on December 20th 1916 he could not walk without assistance, the sole of the left foot was drawn up by spasmodic contraction of the flexor muscles of the front of the leg, and his weight when treading on the left foot was borne entirely on the heel. He was treated by repeatedly extending the foot against the resistance of the contracted muscles. After some hours the spasm of the flexor muscles disappeared and the foot could be moved without resistance as desired. Patient quickly walked quite naturally and was discharged on May 11th, 1917.

Advantages of the treatment are : —

1. The patient can not only generally cooperate in his own cure but later on can in turn help in the treatment of others.

2. No apparatus of any kind is required and no specialized knowledge on the part of the physician.

3. It is accessible to all.

3. It is applicable to cases which have been treated previously without success by other methods.

4. The process by which the contracture is actually eliminated is a physiological one and is not subject to interference from psychical factors, while fear of relapse is reduced by the patient's knowledge of the mechanism of both disability and cure.

